# The Risks Associated With Alcohol Use and Alcoholism

**Published:** 2011

**Authors:** Jürgen Rehm

**Keywords:** alcohol and other drug (AOD) use, alcohol use disorders, alcoholism, heavy drinking, AOD induced risk, AOD effects and consequences, health, disease cause, disease factor, disease risk and protective factors, burden of disease, health care costs, injury, social harm, drinking guidelines, prevention

## Abstract

Alcohol consumption, particularly heavier drinking, is an important risk factor for many health problems and, thus, is a major contributor to the global burden of disease. In fact, alcohol is a necessary underlying cause for more than 30 conditions and a contributing factor to many more. The most common disease categories that are entirely or partly caused by alcohol consumption include infectious diseases, cancer, diabetes, neuropsychiatric diseases (including alcohol use disorders), cardiovascular disease, liver and pancreas disease, and unintentional and intentional injury. Knowledge of these disease risks has helped in the development of low-risk drinking guidelines. In addition to these disease risks that affect the drinker, alcohol consumption also can affect the health of others and cause social harm both to the drinker and to others, adding to the overall cost associated with alcohol consumption. These findings underscore the need to develop effective prevention efforts to reduce the pain and suffering, and the associated costs, resulting from excessive alcohol use.

Alcohol consumption has been identified as an important risk factor for illness, disability, and mortality (Rehm et al. 2009*b*). In fact, in the last comparative risk assessment conducted by the World Health Organization (WHO), the detrimental impact of alcohol consumption on the global burden of disease and injury was surpassed only by unsafe sex and childhood underweight status but exceeded that of many classic risk factors, such as unsafe water and sanitation, hyper-tension, high cholesterol, or tobacco use (WHO 2009). This risk assessment evaluated the net effect of all alcohol consumption—that is, it also took into account the beneficial effects that alcohol consumption (primarily moderate consumption) can have on ischemic diseases^1^ and diabetes (Baliunas et al. 2009; Corrao et al. 2000; Patra et al. 2010; Rehm et al. 2004). Although these statistics reflect the consequences of all alcohol consumption, it is clear that most of the burden associated with alcohol use stems from regular heavier drinking, defined, for instance, as drinking more than 40 grams of pure alcohol per day for men and 20 grams of pure alcohol per day for women^2^ (Patra et al. 2009; Rehm et al. 2004). In addition to the average volume of alcohol consumption, patterns of drinking—especially irregular heavy-drinking occasions, or binge drinking (defined as drinking at least 60 grams of pure alcohol or five standard drinks in one sitting)—markedly contribute to the associated burden of disease and injury (Gmel et al. 2010; Rehm et al. 2004). This article first defines which conditions necessarily are caused by alcohol use and for which conditions alcohol use is a contributing factor. It then looks more closely at the most common disease risks associated with excessive alcohol use, before exploring how these risks have influenced guidelines for drinking limits. The article concludes with a discussion of the alcohol-related risk of harm to people other than the drinker.

## Disease and Injury Conditions Associated With Alcohol Use

### Conditions for Which Alcohol Is a Necessary Cause

More than 30 conditions listed in the WHO’s *International Classification of Diseases, 10th Edition* (ICD–10) (WHO 2007) include the term “alcohol” in their name or definition, indicating that alcohol consumption is a necessary cause underlying these conditions (see table 1). The most important disease conditions in this group are alcohol use disorders (AUDs), which include alcohol dependence and harmful use or alcohol abuse.^3^ AUDs are less fatal than other chronic disease conditions but are linked to considerable disability (Samokhvalov et al. 2010*d*). Overall, even though AUDs in themselves do not rank high as a cause of death globally, they are the fourth-most disabling disease category in low- to middle-income countries and the third-most disabling disease category in high-income countries (WHO 2008). Thus, AUDs account for 18.4 million years of life lost to disability (YLDs), or 3.5 percent of all YLDs, in low- and middle-income countries and for 3.9 million YLDs, or 5.7 percent of all YLDs, in high-income countries. However, AUDs do not affect all population subgroups equally; for example, they mainly affect men, globally representing the second-most disabling disease and injury condition for men. In contrast, AUDs are not among the 10 most important causes of disabling disease and injury in women (WHO 2008).

Alcoholic liver disease and alcohol-induced pancreatitis are other alcohol-specific disease categories that are of global importance. However, no global prevalence data on these disease categories exist because they cannot be validly assessed on a global level. Thus, these conditions are too specific to assess using verbal autopsies and other methods normally used in global-burden-of-disease studies (Lopez et al. 2006; pancreatitis can be estimated indirectly Rajaratnam et al. 2010). Nevertheless, via the prevalence of alcohol exposure the prevalence of alcohol-attributable and relative risk for the wider, unspecific liver cirrhosis and alcohol-induced disease categories (Rehm et al. 2010*a*).

### Conditions for Which Alcohol Is a Component Cause

Disease and injury conditions for which alcohol consumption is a component cause contribute more to the global burden of disease than do alcohol-specific conditions. Overall, the following are the main disease and injury categories impacted by alcohol consumption (listed in the order of their ICD–10 codes):

- Infectious disease;
- Cancer;
- Diabetes;
- Neuropsychiatric disease;
- Cardiovascular disease;
- Liver and pancreas disease; and
- Unintentional and intentional injury.

For all chronic disease categories for which detailed data are available, those data show that women have a higher risk of these conditions than men who have consumed the same amount of alcohol; however, the differences are small at lower levels of drinking (Rehm et al. 2010*a*). The following sections will look at these disease categories individually.

## Individual Disease and Injury Conditions Associated With Alcohol Use

### Infectious Diseases

Although infectious diseases were not included in the WHO’s comparative risk assessments for alcohol conducted in 2000 (Rehm et al. 2004) and 2004 (Rehm et al. 2009*b*), evidence has been accumulating that alcohol consumption has a detrimental impact on key infectious diseases (Rehm et al. 2009*a*, 2010*a*), such as tuberculosis (Lönnroth et al. 2008; Rehm et al. 2009*c*), infection with the human immunodeficiency virus (HIV) (Baliunas et al. 2010; Shuper et al. 2010), and pneumonia (Samokhvalov et al. 2010*c*). In fact, recent studies (Rehm and Parry 2009; Rehm et al. 2009*a*) found that the overall impact of alcohol consumption on infectious diseases is substantial, especially in sub-Saharan Africa.

One of the pathways through which alcohol increases risk for these diseases is via the immune system, which is adversely affected by alcohol consumption, especially heavy drinking (Rehm et al. 2009*c*; Romeo et al. 2010). As a result, although risk for infectious diseases does not differ greatly for people drinking less than 40 grams of pure alcohol per day compared with abstainers, this risk increases substantially for those who drink larger amounts or have been diagnosed with an AUD (Lönnroth et al. 2008; Samokhvalov et al. 2010*c*). In addition, alcohol consumption is associated with poorer outcomes from infectious disease for heavy drinkers by way of social factors. Thus, people with alcohol dependence often are stigmatized and have a higher chance of becoming unemployed and destitute; as a result, they tend to live in more crowded quarters with higher chances for infection and lower chances of recovery (Lönnroth et al. 2009).

The relationship between alcohol consumption and HIV infection and acquired immunodeficiency syndrome (AIDS) is different from that with other infectious diseases. To become infected with HIV, people must exchange body fluids, in most cases either by injecting drugs with a contaminated needle or, more commonly in low-income societies, engaging in unsafe sex. Thus, although significant associations exist between alcohol use, especially heavy drinking, and HIV infection via alcohol’s general effects on the immune system (Baliunas et al. 2010; Kalichman et al. 2007; Shuper et al. 2009, 2010), it cannot be excluded that other variables, including personality characteristics, psychiatric disorders, and situational factors may be responsible for both risky drinking and unsafe sex (Shuper et al. 2010). Researchers frequently have pointed out that personality characteristics, such as a propensity for risk-taking, sensation-seeking, and sexual compulsivity, may be involved in the risk of HIV infection. Indeed, a recent consensus meeting determined that there is not yet sufficient evidence to conclude that alcohol has a causal impact on HIV infection (Parry et al. 2009). However, it can be argued that experimental studies in which alcohol consumption led to a greater inclination to engage in unsafe sex indicate that some causal relationship between alcohol and HIV infection exists (e.g., George et al. 2009; Norris et al. 2009).

Once a person is infected with HIV, alcohol clearly has a detrimental impact on the course of the disease, especially by interfering with effective antiretroviral treatment (Pandrea et al. 2010). A recent meta-analysis found that problem drinking—defined as meeting the National Institute on Alcohol Abuse and Alcoholism (NIAAA)’s criteria for at-risk drinking or having an AUD—was associated with being less than half as likely to adhere to antiretroviral treatment guidelines (Hendershot et al. 2009). Because the level of adherence to the treatment regimen affects treatment success as well as outright survival, alcohol consumption clearly is associated with negative outcomes for people living with HIV and AIDS.

### Cancer

Recently, the Monograph Working Group of the International Agency for Research on Cancer concluded that there was sufficient evidence for the carcinogenicity of alcohol in animals and classified alcoholic beverages as carcinogenic to humans (Baan et al. 2007). In particular, the group confirmed, or newly established, the causal link between alcohol consumption and cancer of the oral cavity, pharynx, larynx, esophagus, liver, colorectum, and female breast. For stomach and lung cancer, carcinogenicity was judged as possible but not established. For all sites where alcohol’s causal role in cancer is established, there is evidence of a dose-response relationship, with relative risk rising linearly with an increasing volume of alcohol consumption (Corrao et al. 2004).

The molecular and biochemical mechanisms by which chronic alcohol consumption leads to the development of cancers of various organs are not fully understood. It has been suggested that these mechanisms differ by target organ and include variations (i.e., polymorphisms) in genes encoding enzymes responsible for ethanol metabolism (e.g., alcohol dehydrogenase, aldehyde dehydrogenase, and cytochrome P450 2E1), increased estrogen concentrations, and changes in folate metabolism and DNA repair (Boffetta and Hashibe 2006; Seitz and Becker 2007). In addition, the International Agency for Research on Cancer group concluded that acetaldehyde—which is produced when the body breaks down (i.e., metabolizes) beverage alcohol (i.e., ethanol) but also is ingested as a component of alcoholic beverages— itself is carcinogenic. It likely plays an important role in the development of cancers of the digestive tract, especially those of the upper digestive tract (Lachenmeier et al. 2009; Seitz and Becker 2007).

### Diabetes

The relationship between alcohol consumption and diabetes is complex. A curvilinear relationship exists between the average volume of alcohol consumption and the inception of diabetes (Baliunas et al. 2009)—that is, lower alcohol consumption levels have a protective effect, whereas higher consumption is associated with an increased risk. The greatest protective effect has been found with a consumption of about two standard drinks (28 grams of pure alcohol) per day, and a net detrimental effect has been found starting at about four standard drinks (50 to 60 grams of pure alcohol) per day.

### Neuropsychiatric Disorders

With respect to neuropsychiatric disorders, alcohol consumption has by far the greatest impact on risk for alcohol dependence. However, alcohol also has been associated with basically all mental disorders (e.g., Kessler et al. 1997), although the causality of these associations is not clear. Thus, mental disorders may be caused by AUDs or alcohol use, AUDs may be caused by other mental disorders, or third variables may be causing both AUDs and other mental disorders. This complex relationship makes it difficult to determine the fraction of mental disorders actually caused by alcohol consumption (see Grant et al. 2009).

The relationship between alcohol and epilepsy is much clearer. There is substantial evidence that alcohol consumption can cause unprovoked seizures, and researchers have identified plausible biological pathways that may underlie this relationship (Samokhvalov et al. 2010*a*). Most of the relevant studies found that a high percentage of heavy alcohol users with epilepsy meet the criteria of alcohol dependence.

### Cardiovascular Diseases

The overall effect of alcohol consumption on the global cardiovascular disease burden is detrimental (see table 2). Cardiovascular disease is a general category that includes several specific conditions, and alcohol’s impact differs for the different conditions. For example, the effect of alcohol consumption on hypertension is almost entirely detrimental, with a dose-response relationship that shows a linear increase of the relative risk with increasing consumption (Taylor et al. 2009). A similar dose-response relationship exists between alcohol consumption and the incidence of atrial fibrillation^4^ (Samokhvalov et al. 2010*b*). On the other hand, for heart disease caused by reduced blood supply to the heart (i.e., ischemic heart disease), the association with alcohol consumption is represented by a J-shaped curve (Corrao et al. 2000), with regular light drinking showing some protective effects. Irregular heavy drinking occasions, however, can nullify any protective effect. In a recent systematic review and meta-analysis comparing the effects of different drinking patterns in people with an overall consumption of less than 60 grams of pure alcohol per day, Roerecke and Rehm (2010) found that consumption of 60 grams of pure alcohol on one occasion at least once a month eliminated any protective effect of alcohol consumption on mortality. The authors concluded that the cardio-protective effect of moderate alcohol consumption disappears when light to moderate drinking is mixed with irregular heavy-drinking occasions. These epidemiological results are consistent with the findings of biological studies that—based on alcohol’s effects on blood lipids and blood clotting—also predict beneficial effects of regular moderate drinking but detrimental effects of irregular heavy drinking (Puddey et al. 1999; Rehm et al. 2003).

The effects of alcohol consumption on ischemic stroke^5^ are similar to those on ischemic heart disease, both in terms of the risk curve and in terms of biological pathways (Patra et al. 2010; Rehm et al. 2010*a*). On the other hand, alcohol consumption mainly has detrimental effects on the risk for hemorrhagic stroke, which are mediated at least in part by alcohol’s impact on hypertension.

Overall, the effects of alcohol consumption on cardiovascular disease are detrimental in all societies with large proportions of heavy-drinking occasions, which is true for most societies globally (Rehm et al. 2003*a*). This conclusion also is supported by ecological analyses or natural experiments. For example, studies in Lithuania (Chenet et al. 2001) found that cardiovascular deaths increased on weekends, when heavy drinking is more common. Also, when overall consumption was reduced in the former Soviet Union (a country with a high proportion of heavy-drinking occasions) between 1984 and 1994, the death rate from cardiovascular disease declined, indicating that alcohol consumption had an overall detrimental effect on this disease category (Leon et al. 1997).

### Diseases of the Liver and Pancreas

Alcohol consumption has marked and specific effects on the liver and pancreas, as evidenced by the existence of disease categories such as alcoholic liver disease, alcoholic liver cirrhosis, and alcohol-induced acute or chronic pancreatitis. For these disease categories, the dose-response functions for relative risk are close to exponential (Irving et al. 2009; Rehm et al. 2010*b*), although the risks associated with light to moderate drinking (i.e., up to 24 grams of pure alcohol per day) are not necessarily different from the risks associated with abstention. Thus, the incidence of diseases of the liver and pancreas is associated primarily with heavy drinking.

It is important to note that given the same amount of drinking, the increase in the risk for mortality from these diseases is greater than the increase in risk for morbidity, especially at lower levels of consumption. This finding suggests that continued alcohol consumption, even in low doses, after the onset of liver or pancreas disease, increases the risk of severe consequences.

### Unintentional Injuries

The link between alcohol and almost all kinds of unintentional injuries has long been established. It depends on the blood alcohol concentration (BAC) and shows an exponential dose-response relationship (Taylor et al. 2010). Alcohol affects psychomotor abilities, with a threshold dose for negative effects generally found at BACs of approximately 0.04 to 0.05 percent (which typically are achieved after consuming two to three drinks in an hour); accordingly, injury resulting from alcohol’s disruption of psychomotor function could occur in people with BACs at this level (Eckardt et al. 1998). However, the epidemiological literature shows that even at lower BACs, injury risk is increased compared with no alcohol consumption (Taylor et al. 2010).

The acute effects of alcohol consumption on injury risk are mediated by how regularly the individual drinks. People who drink less frequently are more likely to be injured or to injure others at a given BAC compared with regular drinkers, presumably because of less tolerance (Gmel et al. 2010). This correlation was demonstrated with respect to traffic injuries in a reanalysis (Hurst et al. 1994) of a classic study conducted in Grand Rapids, Michigan (Borkenstein et al. 1974). It also is important to realize that even if the absolute risk for injury may be relatively small for each occasion of moderate drinking (defined as drinking up 36 grams pure alcohol in one sitting), the lifetime risks from such drinking occasions sums up to a considerable risk for those who often drink at such a level (Taylor et al. 2008).

### Intentional Injuries

Alcohol consumption is linked not only to unintentional but also to intentional injury. Both average volume of alcohol consumption and the level of drinking before the event have been shown to affect suicide risk (Borges and Loera 2010). There also is a clear link between alcohol consumption and aggression, including, but not limited to, homicides (Rehm et al. 2003*b*). Several causal pathways have been identified that play a role in this link, including biological pathways acting via alcohol’s effect on receptors for the brain signaling molecules (i.e., neurotransmitters) serotonin and γ-aminobutyric acid or via alcohol’s effects on cognitive functioning (Rehm et al. 2003*b*). Cultural factors that are related to both differences in drinking patterns and beliefs and expectations about the effects of alcohol also influence the relationship between drinking and aggression (Bushman and Cooper 1990; Graham 2003; Leonard 2005; Room and Rossow 2001).

## Implications of Alcohol-Related Risks for Drinking Guidelines

Overall, the various risks associated with alcohol use at various levels can be combined to derive low-risk drinking guidelines. Such analyses found that overall, any increase in drinking beyond one standard drink on average per day is associated with an increased net risk for morbidity and mortality in high-income countries (Rehm et al. 2009). Moreover, at any given consumption level this risk increase is larger for women than for men. NIAAA has translated the epidemiological findings into low-risk drinking limits of no more than 14 standard drinks per week for men and 7 standard drinks per week for women (NIAAA 2010). These guidelines also specify that to limit the risk of acute consequences, daily consumption should not exceed four standard drinks for men and three for women (NIAAA 2010).

## Overall Global Impact of Alcohol Consumption on Burden of Disease

The most recent systematic overview on the effects of alcohol on global burden of disease was based on data for the year 2004 (Rehm et al. 2009*a*, *b*) (see table 2). The analyses found that although AUDs (which constitute the major part of the neuropsychiatric disorders listed in the table) clearly are important contributors to global burden of disease, they only account for less than one-third of the overall impact of alcohol consumption. Almost equally important are the acute effects of alcohol consumption on the risk of both unintentional and intentional injury. In addition, alcohol has a sizable effect on the burden of disease associated with infectious diseases, cancer, cardiovascular disease, and liver cirrhosis. However, alcohol consumption also has beneficial effects on the burden of disease, mainly on diabetes and the ischemic disease subcategory of cardiovascular diseases. Yet these effects are by far outweighed by the detrimental consequences of alcohol consumption.

## Effects of Alcohol on People Other Than the Drinker

So far, the discussion has centered on alcohol’s effects on health as measured by indicators that primarily are based on the records of hospitals and health systems. Reflecting the information contained in those records, most of the effects considered refer to the health of the drinker. However, this analytic approach omits two large classes of adverse consequences of alcohol: social harm to the drinker and social and health harms to others that result from the drinker’s alcohol consumption. According to the Constitution of the WHO (WHO 1946), health is “a state of complete physical, mental and social well-being and not merely the absence of disease or infirmity” (p. 100); this definition therefore takes into account not just physical and mental harms but also social harms, both for the drinker and for others.

A few examples of harm to others are included in the analysis of alcohol’s contribution to the global burden of disease listed in table 2. These include perinatal conditions attributable to the mother’s drinking during pregnancy and injuries, particularly assault injuries. However, the scope of alcohol-related social harm and of harm to others stretches well beyond these items. Thus, a recent study in Australia (Laslett et al. 2010) identified the following harms to others associated with drinking:

- Harms identified based on records—these included deaths and hospitalizations (e.g., attributed to traffic injuries because of driving under the influence), child abuse or neglect cases involving a caregiver’s drinking, and domestic and other assaults; and
- Harms based on survey reports—these included negative effects on coworkers, household members, other relatives and friends, strangers, and on the community as a whole.

These effects were quite prevalent. Thus, the researchers estimated that within 1 year, more than 350 deaths were attributed to drinking by others, and more than 10 million Australians (or 70 percent of all adults) were negatively affected by a stranger’s drinking (Laslett et al. 2010).

### Social Harm

Drinkers also experience a range of social harms because of their own drinking, including family disruption, problems at the workplace (including unemployment), criminal convictions, and financial problems (Casswell and Thamarangsi 2009; Klingemann and Gmel 2001). Unfortunately, assessment of these problems is much less standardized than assessment of health problems, and many of these harms are not reported continuously. Social-cost studies provide irregular updates of alcohol-attributable consequences in selected countries (for an overview, see Rehm et al. 2009*b*; Thavorncharoensap et al. 2009). These studies regularly find that health care costs comprise only a small portion of the overall costs associated with alcohol use and that most of the alcohol-associated costs are attributable to productivity losses. In total, the costs associated with alcohol use seem to amount to 1 to 3 percent of the gross domestic product in high-income countries; the alcohol-associated costs in South Korea and Thailand, the only two mid-income countries for which similar studies are available, were at about the same level.

## Conclusions

As this review has shown, alcohol use is associated with tremendous costs to the drinker, those around him or her, and society as a whole. These costs result from the increased health risks (both physical and mental) associated with alcohol consumption as well as from the social harms caused by alcohol. To reduce alcohol’s impact on the burden of disease as well as on other social, legal, and monetary costs, it therefore is imperative to develop effective interventions that can prevent or delay initiation of drinking among those who do not drink, particularly adolescents, and limit consumption to low-risk drinking levels among those who do consume alcohol. The remaining articles in this journal issue present several such intervention approaches that are being implemented and evaluated in a variety of settings and/or are targeted at different population subgroups. Together with alcohol-related prevention policies, the implementation of specific interventions with proven effectiveness can help reduce the pain and suffering, and the associated costs, resulting from excessive alcohol use.

## Figures and Tables

**Table 1:** Disease Conditions That by Definition Are Attributable to Alcohol (AAF = 100%)

**ICD–10 Code**	**Disease**
E24.4	Alcohol-induced pseudo-Cushing’s syndrome
F10	Mental and behavioral disorders attributed to use of alcohol
*F10.0*	*Acute intoxication*
*F10.1*	*Harmful use*
*F10.2*	*Dependence syndrome*
*F10.3*	*Withdrawal state*
*F10.4*	*Withdrawal state with delirium*
*F10.5*	*Psychotic disorder*
*F10.6*	*Amnesic syndrome*
*F10.7*	*Residual and late-onset psychotic disorder*
*F10.8*	*Other mental and behavioral disorders*
*F10.9*	*Unspecified mental and behavioral disorder*
G31.2	Degeneration of nervous system attributed to alcohol
G62.1	Alcoholic polyneuropathy
G72.1	Alcoholic myopathy
I42.6	Alcoholic cardiomyopathy
K29.2	Alcoholic gastritis
K70	Alcoholic liver disease
*K70.0*	*Alcoholic fatty liver*
*K70.1*	*Alcoholic hepatitis*
*K70.2*	*Alcoholic fibrosis and sclerosis of liver*
*K70.3*	*Alcoholic cirrhosis of liver*
*K70.4*	*Alcoholic hepatic failure*
*K70.9*	*Alcoholic liver disease, unspecified*
K85.2	Alcohol-induced acute pancreatitis
K86.0	Alcohol-induced chronic pancreatitis
O35.4	Maternal care for (suspected) damage to fetus from alcohol
P04.3	Fetus and newborn affected by maternal use of alcohol
Q86.0	Fetal alcohol syndrome (dysmorphic)
R78.0	Finding of alcohol in blood
T51	Toxic effect of alcohol
*T51.0*	*Ethanol*
*T51.1*	*Methanol*
*T51.8*	*Other alcohols*
*T51.9*	*Alcohol unspecified*
X45	Accidental poisoning by and exposure to alcohol
X65	Intentional self-poisoning by and exposure to alcohol
Y15	Poisoning by and exposure to alcohol, undetermined intent
Y90	Evidence of alcohol involvement determined by blood alcohol level

Note: ICD codes in italics represent subcodes within a main code of classification.

Abbreviations: AAF = alcohol-attributable fraction.

**Table 2:** Global Burden of Alcohol-Attributable Disease in Disability-Adjusted Life Years (DALYs) (in 1,000s) by Sex and Disease Category for the Year 2004

**Disease Category**	**World**					
	**M**	**W**	**T**	**%M**	**%W**	**%**
Infectious disease	7,057	1,186	8,243	10.2	9.5	10.1
Maternal and perinatal conditions (low birth weight)	64	55	119	0.1	0.4	0.1
Cancer	4,732	1,536	6,268	6.9	12.3	7.7
Diabetes	0^*^	28	28	0.0	0.2	0.0
Neuropsychiatric disorders	23,265	3,417	26,682	33.7	27.3	32.7
Cardiovascular diseases	5,985	939	6,924	8.7	7.5	8.5
Cirrhosis of the liver	5,502	1,443	6,945	8.0	11.5	8.5
Unintentional injuries	15,694	2,910	18,604	22.8	23.2	22.8
Intentional injuries	6,639	1,021	7,660	9.6	8.1	9.4
**Total detrimental effects attributable to alcohol**	68,938	12,536	81,474	100.0	100.0	100.0
Diabetes	−238	−101	−340	22.2	8.1	14.6
Cardiovascular diseases	−837	−1,145	−1,981	77.8	91.9	85.4
**Total beneficial effects attributable to alcohol**	−1,075	−1,246	−2,321	100.0	100.0	100.0
**All alcohol-attributable net DALYs**	**67,863**	**11,290**	**79,153**			
**All DALYs**	799,536	730,631	1,530,168			
**Percentage of all net DALYs attributable to alcohol**	**8.5%**	**1.5%**	**5.2%**			
**For comparison without infectious disease**	**7.6%**	**1.4%**	**4.6%**			

NOTE: M = men; W = women; T = total.

* Numbers are rounded to the nearest thousand. Zero (0) indicates that fewer than 500 alcohol-attributable DALYs in the disease category.

SOURCE: Rehm et al. 2009*a,b*.

## Footnotes

1 Ischemic diseases are all conditions that are related to the formation of blood clots, which prevent adequate blood flow to certain tissues.

2 In the United States, a standard drink usually is considered to contain 0.6 fluid ounces (or 14 grams) of pure alcohol. This is the amount of ethanol found in approximately 12 ounces of beer, 5 ounces of wine, or 1.5 ounces of distilled spirits. However, many drinks, as actually poured, contain more alcohol. Thus, for example, a glass of wine often contains more than 5 fluid ounces and therefore may correspond to one and a half or even two standard drinks.

3 The condition referred to as “harmful use” in the ICD–10 loosely corresponds to “alcohol abuse,” as defined in the American Psychiatric Association’s *Diagnostic and Statistical Manual of Mental Diseases, 4th Edition* (DSM–IV).

4 Atrial fibrillation is an abnormal heart rhythm involving the two upper chambers (i.e., atria) of the heart.

5 A stroke is the disruption of normal blood flow to a brain region. In the case of an ischemic stroke, this is caused by blockage of a blood vessel that prevents the blood from reaching neighboring brain areas. In the case of a hemorrhagic stroke, rupture of a blood vessel and bleeding into the brain occurs, which prevents normal blood supply to other brain regions.

## References

[b1-arh-34-2-135] Baan R, Straif K, Grosse Y (2007). Carcinogenicity of alcoholic beverages. Lancet Oncology.

[b2-arh-34-2-135] Baliunas D, Rehm J, Irving H, Shuper P (2010). Alcohol consumption and risk of incident human immunodeficiency virus infection: A meta-analysis. International Journal of Public Health.

[b3-arh-34-2-135] Baliunas DO, Taylor BJ, Irving H (2009). Alcohol as a risk factor for type 2 diabetes: A systematic review and meta-analysis. Diabetes Care.

[b4-arh-34-2-135] Boffetta P, Hashibe M (2006). Alcohol and cancer. Lancet Oncology.

[b5-arh-34-2-135] Borges G, Loera CR (2010). Alcohol and drug use in suicidal behaviour. Current Opinion in Psychiatry.

[b6-arh-34-2-135] Borkenstein RF, Crowther FR, Shumate RP (1974). The role of the drinking driver in traffic accidents (The Grand Rapids Study). Blutalkohol.

[b7-arh-34-2-135] Bushman BJ, Cooper HM (1990). Effects of alcohol on human aggression: An integrative research review. Psychological Bulletin.

[b8-arh-34-2-135] Casswell S, Thamarangsi T (2009). Reducing the harm from alcohol: Call to action. Lancet.

[b9-arh-34-2-135] Chenet L, Britton A, Kalediene R, Petrauskiene J (2001). Daily variations in deaths in Lithuania: The possible contribution of binge drinking. International Journal of Epidemiology.

[b10-arh-34-2-135] Corrao G, Bagnardi V, Zambon A, La Vecchia C (2004). A meta-analysis of alcohol consumption and the risk of 15 diseases. Preventive Medicine.

[b11-arh-34-2-135] Corrao G, Rubbiati L, Bagnardi V (2000). Alcohol and coronary heart disease: A meta-analysis. Addiction.

[b12-arh-34-2-135] Eckardt MJ, File SE, Gessa GL (1998). Effects of moderate alcohol consumption on the central nervous system. Alcoholism: Clinical and Experimental Research.

[b13-arh-34-2-135] George WH, Davis KC, Norris J (2009). Indirect effects of acute alcohol intoxication on sexual risk-taking: The roles of subjective and physiological sexual arousal. Archives of Sexual Behavior.

[b14-arh-34-2-135] Gmel G, Kuntsche E, Rehm J (2010). Risky single occasion drinking: Bingeing is not bingeing. Addiction.

[b15-arh-34-2-135] Graham K, Mattson M (2003). Social drinking and aggression. Neurobiology of Aggression: Understanding and Preventing Violence.

[b16-arh-34-2-135] Grant BF, Goldstein RB, Chou SP (2009). Sociodemographic and psychopathologic predictors of first incidence of DSM-IV substance use, mood and anxiety disorders: Results from the Wave 2 National Epidemiologic Survey on Alcohol and Related Conditions. Molecular Psychiatry.

[b17-arh-34-2-135] Hendershot CS, Stoner SA, Pantalone DW, Simoni JM (2009). Alcohol use and antiretroviral adherence: Review and meta-analysis. Journal of Acquired Immune Deficiency Syndromes.

[b18-arh-34-2-135] Hurst PM, Harte D, Firth WJ (1994). The Grand Rapids dip revisited. Accident: Analysis and Prevention.

[b19-arh-34-2-135] Irving HM, Samokhvalov AV, Rehm J (2009). Alcohol as a risk factor for pancreatitis. A systematic review and meta-analysis. Journal of the Pancreas.

[b20-arh-34-2-135] Kalichman SC, Simbayi LC, Kaufman M (2007). Alcohol use and sexual risks for HIV/AIDS in sub-Saharan Africa: Systematic review of empirical findings. Prevention Science.

[b21-arh-34-2-135] Kessler RC, Crum RM, Warner LA (1997). Lifetime co-occurrence of DSM-III-R alcohol abuse and dependence with other psychiatric disorders in the National Comorbidity Survey. Archives of General Psychiatry.

[b22-arh-34-2-135] Klingemann H, Gmel G (2001). Mapping Social Consequences of Alcohol Consumption.

[b23-arh-34-2-135] Lachenmeier DW, Kanteres F, Rehm J (2009). Carcinogenicity of acetaldehyde in alcoholic beverages: Risk assessment outside ethanol metabolism. Addiction.

[b24-arh-34-2-135] Laslett AM, Catalano P, Chikritzhs T (2010). The Range and Magnitude of Alcohol’s Harm to Others.

[b25-arh-34-2-135] Leon DA, Chenet L, Shkolnikov VM (1997). Huge variation in Russian mortality rates 1984– 1994. Artefact, alcohol, or what?. Lancet.

[b26-arh-34-2-135] Leonard KE (2005). Alcohol and intimate partner violence: When can we say that heavy drinking is a contributing cause of violence?. Addiction.

[b27-arh-34-2-135] Lönnroth K, Jaramillo E, Williams BG (2009). Drivers of tuberculosis epidemics: The role of risk factors and social determinants. Social Science & Medicine.

[b28-arh-34-2-135] Lönnroth K, Williams BG, Stadlin S (2008). Alcohol use as a risk factor for tuberculosis: A systematic review. BMC Public Health.

[b29-arh-34-2-135] Lopez AD, Mathers CD, Ezzati M (2006). Global Burden of Disease and Risk Factors.

[b30-arh-34-2-135] National Institute on Alcohol Abuse and Alcoholism (NIAAA) (2010). Rethinking Drinking: Alcohol and Your Health.

[b31-arh-34-2-135] Norris J, Stoner SA, Hessler DM (2009). Cognitive mediation of alcohol’ s effects on women’s in-the-moment sexual decision making. Health Psychology.

[b32-arh-34-2-135] Pandrea I, Happel KI, Amedee AM (2010). Alcohol’s Role in HIV Transmission and Disease Progression.

[b33-arh-34-2-135] Parry C, Rehm J, Poznyak V, Room R (2009). Alcohol and infectious diseases: An overlooked causal linkage?. Addiction.

[b34-arh-34-2-135] Patra J, Taylor B, Irving H (2010). Alcohol consumption and the risk of morbidity and mortality from different stroke types: A systematic review and meta-analysis. BMC Public Health.

[b35-arh-34-2-135] Patra J, Taylor B, Rehm J (2009). Deaths associated with high-volume drinking of alcohol among adults in Canada in 2002: A need for primary care intervention?. Contemporary Drug Problems.

[b36-arh-34-2-135] Puddey IB, Rakic V, Dimmitt SB, Bellin LJ (1999). Influence of pattern of drinking on cardiovascular disease and cardiovascular risk factors: A review. Addiction.

[b37-arh-34-2-135] Rajaratnam JK, Marcus JR, Levin-Rector A (2010). Worldwide mortality in men and women aged 15-59 years from 1970 to 2010: A systematic analysis. Lancet.

[b38-arh-34-2-135] Rehm J, Parry C (2009). Alcohol consumption and infectious diseases in South Africa. Lancet.

[b39-arh-34-2-135] Rehm J, Anderson P, Kanteres F (2009a). Alcohol, Social Development and Infectious Disease.

[b40-arh-34-2-135] Rehm J, Baliunas D, Borges GL (2010a). The relation between different dimensions of alcohol consumption and burden of disease: An overview. Addiction.

[b41-arh-34-2-135] Rehm J, Mathers C, Popova S (2009b). Global burden of disease and injury and economic cost attributable to alcohol use and alcohol-use disorders. Lancet.

[b42-arh-34-2-135] Rehm J, Rehn N, Room R (2003a). The global distribution of average volume of alcohol consumption and patterns of drinking. European Addiction Research.

[b43-arh-34-2-135] Rehm J, Room R, Graham K (2003b). The relationship of average volume of alcohol consumption and patterns of drinking to burden of disease: An overview. Addiction.

[b44-arh-34-2-135] Rehm J, Room R, Monteiro M (2004). Alcohol use. Comparative Quantification of Health Risks: Global and Regional Burden of Disease Attributable to Selected Major Risk Factors.

[b45-arh-34-2-135] Rehm J, Samokhvalov AV, Neuman MG (2009c). The association between alcohol use, alcohol use disorders and tuberculosis (TB): A systematic review. BMC Public Health.

[b46-arh-34-2-135] Rehm J, Sempos C, Trevisan M (2003c). Alcohol and cardiovascular disease—More than one paradox to consider. Average volume of alcohol consumption, patterns of drinking and risk of coronary heart disease: A review. Journal of Cardiovascular Risk.

[b47-arh-34-2-135] Rehm J, Taylor B, Mohapatra S (2010b). Alcohol as a risk factor for liver cirrhosis: A systematic review and meta-analysis. Drug and Alcohol Review.

[b48-arh-34-2-135] Rehm J, Zatonski W, Taylor B (2011). Epidemiology and alcohol policy in Europe. Addiction.

[b49-arh-34-2-135] Roerecke M, Rehm J (2010). Irregular heavy drinking occasions and risk of ischemic heart disease: A systematic review and meta-analysis. American Journal of Epidemiology.

[b50-arh-34-2-135] Romeo J, Warnberg J, Marcos A (2010). Drinking pattern and socio-cultural aspects on immune response: An overview. Proceedings of the Nutrition Society.

[b51-arh-34-2-135] Room R, Rossow I (2001). The share of violence attributable to drinking. Journal of Substance Use.

[b52-arh-34-2-135] Samokhvalov AV, Irving H, Mohapatra S, Rehm J (2010a). Alcohol consumption, unprovoked seizures and epilepsy: A systematic review and meta-analysis. Epilepsia.

[b53-arh-34-2-135] Samokhvalov AV, Irving HM, Rehm J (2010b). Alcohol as a risk factor for atrial fibrillation: A systematic review and meta-analysis. European Journal of Cardiovascular Prevention & Rehabilitation.

[b54-arh-34-2-135] Samokhvalov AV, Irving HM, Rehm J (2010c). Alcohol consumption as a risk factor for pneumonia: A systematic review and meta-analysis. Epidemiology and Infection.

[b55-arh-34-2-135] Samokhvalov AV, Popova S, Room R (2010d). Disability associated with alcohol abuse and dependence. Alcoholism: Clinical and Experimental Research.

[b56-arh-34-2-135] Seitz HK, Becker P (2007). Alcohol metabolism and cancer risk. Alcohol Research & Health.

[b57-arh-34-2-135] Shuper PA, Joharchi N, Irving H, Rehm J (2009). Alcohol as a correlate of unprotected sexual behavior among people living with HIV/AIDS: Review and meta-analysis. AIDS and Behavior.

[b58-arh-34-2-135] Shuper PA, Neuman M, Kanteres F (2010). Causal considerations on alcohol and HIV/AIDS: A systematic review. Alcohol and Alcoholism.

[b59-arh-34-2-135] Taylor B, Irving HM, Baliunas D (2009). Alcohol and hypertension: Gender differences in dose-response relationships determined through systematic review and meta-analysis. Addiction.

[b60-arh-34-2-135] Taylor B, Irving HM, Kanteres F (2010). The more you drink, the harder you fall: A systematic review and meta-analysis of how acute alcohol consumption and injury or collision risk increase together. Drug and Alcohol Dependence.

[b61-arh-34-2-135] Taylor B, Rehm J, Room R (2008). Determination of lifetime injury mortality risk in Canada in 2002 by drinking amount per occasion and number of occasions. American Journal of Epidemiology.

[b62-arh-34-2-135] Thavorncharoensap M, Teerawattananon Y, Yothasamut J (2009). The economic impact of alcohol consumption: A systematic review. Substance Abuse Treatment, Prevention, and Policy.

[b63-arh-34-2-135] World Health Organization (WHO) http://www.who.int/about/definition/en/print.html.

[b64-arh-34-2-135] WHO (2007). International Classification of Diseases and Related Health Problems.

[b65-arh-34-2-135] WHO (2008). The Global Burden of Disease: 2004 Update.

[b66-arh-34-2-135] WHO (2009). Global Health Risks. Mortality and Burden of Disease Attributable to Selected Major Risks.

